# Supplementary figures and images for: Pathways to service access for pre-eclampsia and eclampsia in rural Bangladesh: Exploring women’s care-seeking
Source: PLoS One. 2021 Feb 4;16(2):e0245371. doi: 10.1371/journal.pone.0245371 (PMC7861535; doi:10.1371/journal.pone.0245371)

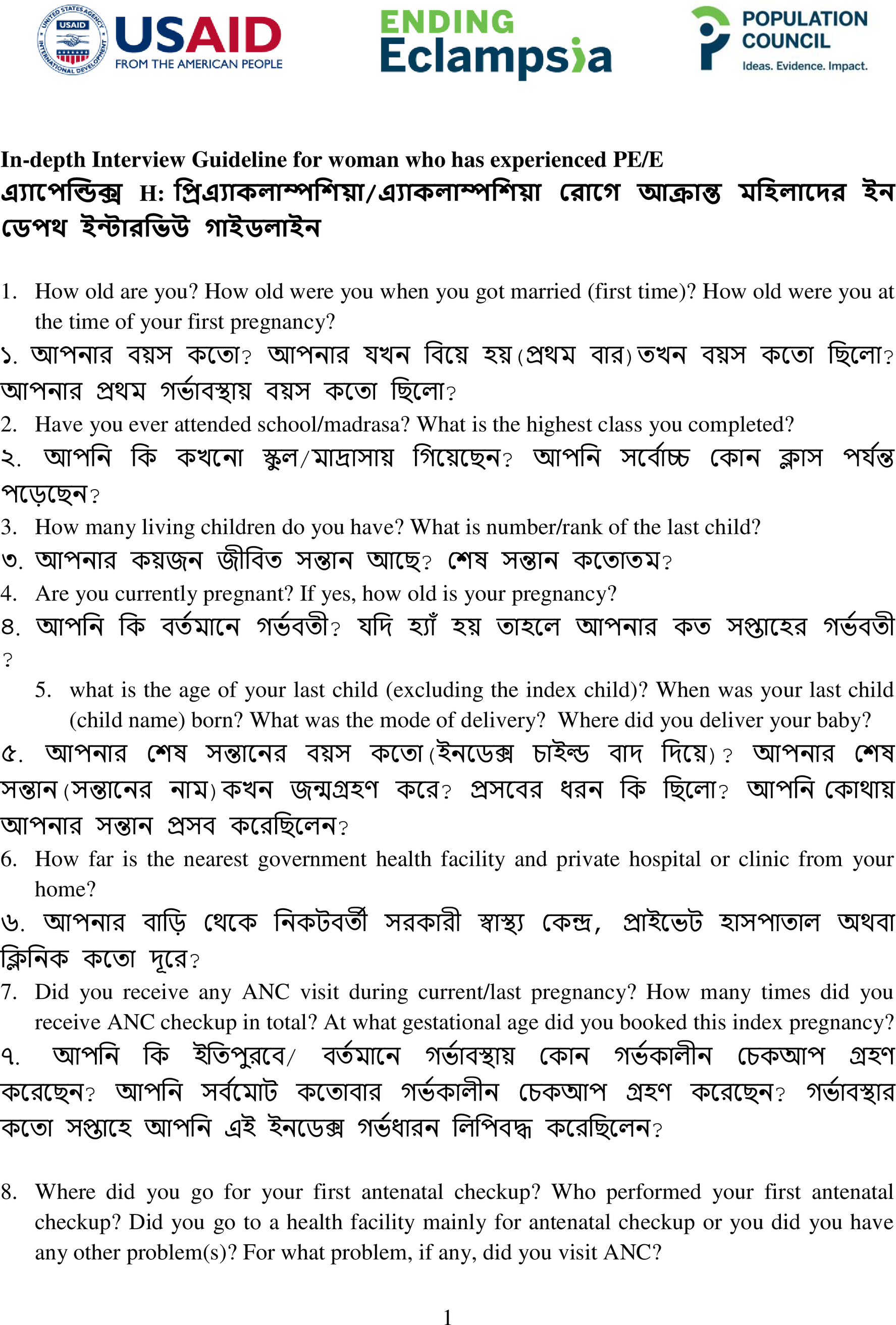

Supplement: S1 Appendix — (TIF) [file pone.0245371.s001.tif]

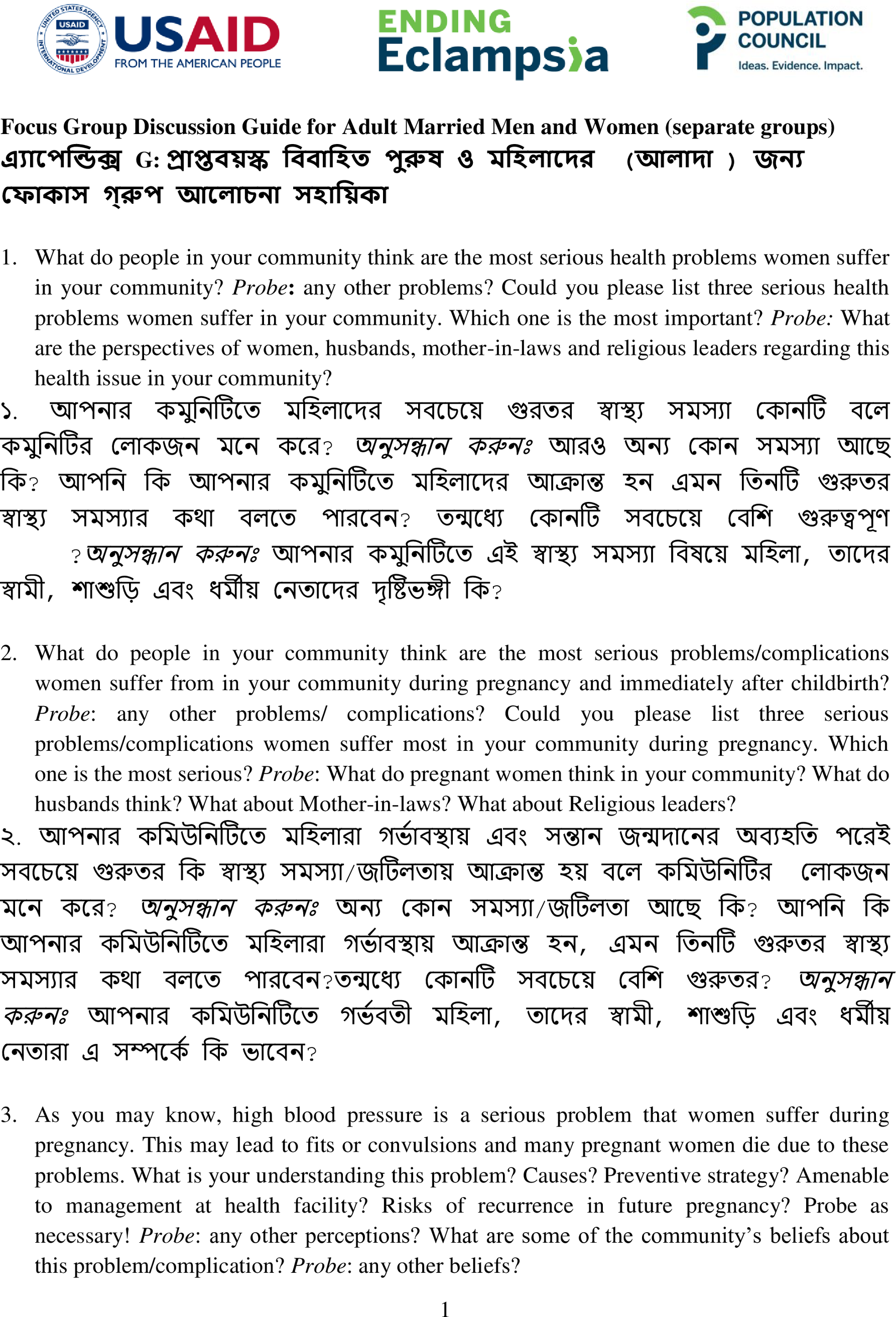

Supplement: S2 Appendix — (TIF) [file pone.0245371.s002.tif]
