# Supplementary material for: Pathways to service access for pre-eclampsia and eclampsia in rural Bangladesh: Exploring women’s care-seeking
Source: PLoS One. 2021 Feb 4;16(2):e0245371. doi: 10.1371/journal.pone.0245371 (PMC7861535; doi:10.1371/journal.pone.0245371)
Supplement: Attachment — Submitted filename: Response to Reviewers.pdf [file pone.0245371.s004.pdf]

POPULATION COUNCIL
Ideas. Evidence. Impact.

…pulation Council
…W, Suite 280
…C 20008
…il.org

Dr. Joerg Hever
Editor in Chief, PLOS ONE
1160 Battery Street
Koshland Building East, Suite 225
San Francisco, CA 94111

October 1, 2020

Dear Dr. Hever,

Thank you for the review of the manuscript entitled, “Pathways to service access for pre-eclampsia and eclampsia in rural Bangladesh: exploring women’s care-seeking.” We hope this revised manuscript is acceptable for publication in PLOS ONE.

We are grateful for the reviewers’ comments and are glad they feel the presentation of pre-eclampsia and eclampsia survivors’ experiences shared in this manuscript is clear and powerful. The focus on these women’s personal stories of accessing maternal health services while experiencing pregnancy-related complications is imperative for improving community perceptions of formal health systems and increasing access to lifesaving care. We welcome the chance to continue improving our manuscript. Please find below detailed responses to the reviewer’s comments and descriptions of corresponding revisions made in the resubmitted manuscript.

This manuscript comes from original work and is not published or under consideration for publication elsewhere. The authors have no competing interests, and we have approved the manuscript for submission

We thank you for your consideration and look forward to hearing from you.

Sincerely,

Amy Dempsey

Amy Dempsey, MA

Population Council
4301 Connecticut Ave NW, Suite 280
Washington, DC 20008

Comment: The correct term is "convenience sample" not "convenient sampling" (even though this makes more grammatical sense).

Response: We have changed this to "were from a convenience sample"

Comment: The following sentence is awkward (pp. 14-15): "Our findings align with other studies that examined maternal healthcare seeking among adolescent girls in Bangladesh, suggesting that women's lack of knowledge of signs and symptoms and the (15) importance of ANC may have a larger interactive effect with internal processing of decisions among younger women (14), as well as socio-economic status (15).Note: You do not indicate what the effect is of the interaction with socioeconomic status, so I'm guessing that it compounds disadvantage. Please correct as needed.

Response: We have changed this to say "Our findings align with other studies that examined maternal healthcare seeking behavior among adolescent girls in Bangladesh, suggesting that women's lack of knowledge about the importance of ANC and its signs and symptoms may negatively interact with socio-economic status of women (15) and affect internal processing of decisions among younger women (14)."

Comment: This sentence would be better placed in the methodology, rather than the conclusion. It is a clarification, not a limitation. "During our IDIs with PE/E survivors, there were two instances where the survivor could not recall the details of her PE/E complications. Therefore, a companion who was present when the survivor experienced those complications and was present during the IDI was able to communicate to researchers the details of the survivor's experience.

Response: Thank you for this comment. This sentence was in both places already - methodology (page 6) and limitations (page 16).

Comments 4 & 5:

4. (a) There is mention of gendered roles in the findings, but no real discussion, until the conclusion. What, specifically, could be done to assist men to make better decisions?

4. (b) The same occurs with lower levels of education: you present it, but do not discuss it. However, you mentions that some women were aware of E/PE through brochures; could public health information help, even for less-educated women?

5. On p. 3, you outlines the following…
"In addition to clinical and health systems issues that negatively impact pregnant women and their families, deaths from PE/E occur largely as a result of delays in seeking health services (4)andthe use of one or more health systems (5,6). Thaddeus and Maine posit that delays in seeking care can occur at three different points: (1) delay in deciding to seek care, (2) delay in arriving at a health facility, and (3) delay in receiving quality care at a facility (7). This paper focuses primarily on the first and second delays, a…

Suggestion: In the conclusion, can you reflect back and connect your results to these propositions? It would make your paper stronger if you contextualized your findings with others' prior studies. You have some evidence, as well, that proposition three is confirmed; that there is delay in receiving services at some facilities, either due to inconsistent service or pay barriers. This seems to be complicated by the involvement of nonmedical staff as gatekeepers.

POPULATION COUNCIL
Ideas. Evidence. Impact.

Response to comments 4 - 5: We have updated this section to say the following:

This study shows variable pathways to care in rural Bangladesh and these pathways are highly influenced by internal and external factors that can be enabling or constraining. Our findings show that deaths from PE/E occur largely as a result of using one or more health systems and delays in seeking health services. The delays most common in our findings are a delay in deciding to seek care and arriving at a health facility. Receiving poor quality care at a facility was also reported, but was not as common in our sample. In addition to these delays, our findings indicate that accessing life-saving maternal health services is a complex process that may be complicated by the many players involved, such as non-medical staff at health facilities.

Pathways were compared across the four districts, and showed consistency in terms of contact points and type of facilities. These consistencies suggest the need to support quality improvement in primary healthcare and strengthen gender equity through community-based health promotion that raises awareness of the importance of birth preparedness and enhance women's autonomy and allows them to make decisions about their reproductive and maternal health care. These community-based health promotion activities can include targeted policy and programming that incorporates women's and men's health groups, religious leaders - trusted community members - and public health information in facilities that utilizes visual imagery of signs and symptoms of complications.

Comment: In the introduction, you state that: "...[in] Bangladesh, between 5,000 and 6,000 women die during pregnancy or childbirth, and up to 1,200 –or 24percent –of those deaths are from eclampsia (10), the second most common cause of maternal mortality after postpartum hemorrhage."

Suggestion: Can you reflect back, in your conclusion, on how much of a difference it might make to tackle various problems? For example, is the primary barrier to better treatment the inability of facilities to provide care? Or is the primary problem lack of knowledge among men (husbands and fathers) and women (mothers, mothers-in-law, etc.) about E/PE; or is the primary problem the circling back and forth between different types of providers (traditional healers, local health clinics, etc)? Your reflections might not be strictly evidence, but you know your material better than anyone else – what are your thoughts?

Response: We have updated this section to say: There is no single barrier to accessing maternal health services, but rather multiple barriers that include social barriers within communities, as well as barriers within individual households and health systems. Each of these play a role in women's ability to access life-saving care.

Comment: Question: How different is this situation than it is in urban Bangladesh? Are these problems of lack of access and knowledge purely rural? I suspect not, so in that case, how can these findings help those in urban areas? Should this be investigated further? Could you make a recommendation for further research?

Response: Thank you for your comment. This has been addressed already in the limitations section.

Comment: Your findings will be of interest to rural studies. Please consider using "rural" as a keyword.

Response: Thank you - we've added this as a keyword.
