# Supplementary material for: Pathways to service access for pre-eclampsia and eclampsia in rural Bangladesh: Exploring women’s care-seeking
Source: PLoS One. 2021 Feb 4;16(2):e0245371. doi: 10.1371/journal.pone.0245371 (PMC7861535; doi:10.1371/journal.pone.0245371)
Supplement: Attachment — Submitted filename: Response to Reviewers.pdf [file pone.0245371.s006.pdf]

POPULATION
COUNCIL
Ideas. Evidence. Impact.

Dr. Joerg Hever
Editor in Chief, PLOS ONE
1160 Battery Street
Koshland Building East, Suite 225
San Francisco, CA 94111

November 13, 2020

Dear Dr. Hever,

Thank you for the review of the manuscript entitled, "Pathways to service access for pre-eclampsia and eclampsia in rural Bangladesh: exploring women's care-seeking." We hope this revised manuscript is acceptable for publication in PLOS ONE.

We are grateful for the reviewers' comments and are glad they feel the presentation of pre-eclampsia and eclampsia survivors' experiences shared in this manuscript is clear and powerful. The focus on these women's personal stories of accessing maternal health services while experiencing pregnancy-related complications is imperative for improving community perceptions of formal health systems and increasing access to lifesaving care. We welcome the chance to continue improving our manuscript. Please find below detailed responses to the reviewer's comments and descriptions of corresponding revisions made in the resubmitted manuscript.

This manuscript comes from original work and is not published or under consideration for publication elsewhere. The authors have no competing interests, and we have approved the manuscript for submission

We thank you for your consideration and look forward to hearing from you.

Sincerely,

Amy Dempsey

Amy Dempsey, MA

Population Council
4301 Connecticut Ave NW, Suite 280
Washington, DC 20008

The Population Council conducts research and delivers solutions that improve lives around the world.
Big ideas supported by evidence: It's our model for global change.

Reviewer Comment: Please note specific typographical issues in the uploaded document. In particular, the references need to be standardized. I have one further substantive question: I notice that in your interview and discussion group questions you ask about the mother-in-law's influence, but not the mother's. I suspect that this is according to Eastern traditions, where the wife goes to live with the husband's family. If this is the case, can you please note that interviewees were not asked about the mother's influence, according to cultural family forms where the wife lives with the husband's family? Western audiences may need assistance to recognize the importance of the mother-in-law relationship.

Response: It seems that the typos were in the quotes from the IDIs and the FGDs. We had previously decided to leave those unedited to ensure the “voice” of the speaker remained. However, once receiving this comment, we have gone through the text and edited for better English.

Re: the references, authors have gone through the reference list and reformatted it.

The authors have added a sentence on page 12 to say: *After marriage, it is common for a woman to move into her husband’s parents’ home, and as a result, mothers-in-law also influence women’s health-seeking practices.*
